# A One-Session Treatment of PTSD After Single Sexual Assault Trauma. A Pilot Study of the WONSA MLI Project: A Randomized Controlled Trial

**DOI:** 10.1177/0886260520965973

**Published:** 2020-10-21

**Authors:** Gita Rajan, Caroline Wachtler, Sara Lee, Per Wändell, Björn Philips, Lars Wahlström, Carl Göran Svedin, Axel C. Carlsson

**Affiliations:** 1 Karolinska Institutet, Huddinge, Sweden; 2 Academic Primary Healthcare Centre, Stockholm, Sweden; 3 Svenska Kognitiva Institutet, Stockholm, Sweden; 4 Stockholm University, Stockholm, Sweden; 5 Ersta Sköndal Bräcke University College, Stockholm, Sweden

**Keywords:** sexual abuse, rape, sexual assault, PTSD, treatment: IES-R, Impact of Event Scale

## Abstract

Sexual abuse is a crime with devastating health consequences. Accessible, acceptable and affordable treatment of PTSD after sexual abuse is important. In this pilot study, a one-session PTSD treatment and a modified perspective to PTSD treatment is introduced. The aim of the study was to test the efficacy of one session of Modified Lifespan Integration (MLI) on reduction of symptoms of PTSD in individuals with PTSD after one sexual assault. This was a single-center, individually randomized waitlist-controlled treatment study with 1:1 allocation, with the intervention of one 90 - 140 minutes session of MLI and with post-treatment follow-up at 3 weeks (time point two). All participants were females, mean age 24, with PTSD symptoms after one sexual assault during the past 5 years. Exclusion criteria were poor understanding of Swedish, multiple traumas, active substance abuse, active psychosis, ADHD, or autism spectrum disorder. Of 135 interested participants, 38 were finally included, 36 completed baseline measures and were included in the intent to treat analyses and 33 were analyzed per protocol. The primary outcome was the difference between the two trial arms in mean PTSD symptoms as measured by the Impact of Event Scale Revised (IES-R) at time point two. In the intervention arm, 72% no longer scored PTSD in per-protocol analysis, compared to 6% in the waiting list arm. IES-R scores were on average halved in the intervention arm (F=21.37, P<0.001), but were essentially unchanged in the waiting list arm. No adverse effects or drop-outs were seen. One session of Modified Lifespan Integration was an effective treatment with a low drop-out rate for females aged 15-65 with PTSD after one sexual assault. Provided that this result can be replicated, MLI should be offered to these patients in clinical settings. Registration number NCT03141047 was given 03/25/2016 at ClinicalTrials.gov (https://register.clinicaltrials.gov/).

## Introduction

The United Nations Sustainable Development Goals specifically target sexual abuse because of its high cross-cultural prevalence and severe harms (United Nations General Assembly, 2015, 5.2 and 16.2). The high incidence (> 50%) of posttraumatic stress disorder (PTSD) after sexual assault makes sexual assault one of the most traumatic experiences a person can be exposed to (Elklit & Christiansen, 2010; Kessler et al., 1995; Masho & Ahmed, 2007; Tiihonen Moller et al., 2014) and might partly explain the high burden of disease among this group (Brady et al., 2000; Greene et al., 2016; Hailes et al., 2019). Considering a prevalence of child sexual abuse of 18.0% (range 11.3–21.5%) among women (Fedina et al., 2018; Stoltenborgh et al., 2011), the risk for PTSD after sexual abuse and the risk for re-victimization and further traumatization after sexual abuse (Finkelhor et al., 2007; Moller et al., 2017; Papalia et al., 2017), an accessible and cost-effective treatment for PTSD is of great importance.

A recent systematic literature review of PTSD treatment, including 64 trials, showed support for exposure-based cognitive behavioral therapy (CBT), including both prolonged exposure (PE), and cognitive processing therapy (CPT), eye movement desensitization and reprocessing (EMDR), and narrative exposure therapy (Cusack et al., 2016; Foa, Gillihan, et al., 2013; Foa & McLean, 2016; Langkaas et al., 2017; Morkved et al., 2014; Raabe et al., 2015). There is also support for imagery rescripting (ImRs) and stress inoculation training (SIT; Foa et al., 1999; Langkaas et al., 2017; Raabe et al., 2015). Most exposure-based therapies use the concept of emotional processing theory (EPT) (Foa & Kozak, 1986), that is, they use interventions for activation of fear structure, habituation, and disconfirmation of erroneous cognitions and beliefs to treat PTSD (Foa & McLean, 2016). However, even for single trauma, traditional exposure-based treatment programs are time consuming, require 8–12 sessions with daily homework, and are therefore both costly and demanding. Even more important, the dropout rates for PTSD treatment are often high in real clinical settings as opposed to the clinical trial settings (Najavits, 2015); even in study settings however, meta-analysis finds dropout rates of between 16 and 18% (Cooper & Conklin, 2015; Imel et al., 2013; Lewis et al., 2020). Attempts to increase the efficacy of trauma treatment by combining different efficacious methods, such as PE and cognitive restructuring (CR) and PE/SIT, have been made in different studies, without enhanced outcomes (Foa et al., 1999; Foa et al., 2005; Marks et al., 1998).

In 2002, Peggy Pace started to develop an approach to trauma treatment not typically cognitive behavioral nor psychodynamic, after having observed that recovery from PTSD was aided when patients identified and visualized episodic memories (memory cues [MCs]) through a chronological timeline for each year since the index trauma up to the present time (Catherine Thorpe, 2012; Pace). The method developed was named Lifespan Integration (LI). In the theory of LI, as defined in this first published article of the method, it is hypothesized that: *The core of the PTSD symptoms are due to a failure of the index trauma to anchor as an episodic memory in the traumatized individual’s chronologic autobiographic memory.* Subsequently, as the index trauma is transformed into an episodic memory anchored in a chronological timeline, the limbic system stops perceiving the index trauma as a potential threat in present time, intrusion and hypervigilance stop, avoidance is no longer needed, and the cardinal symptoms of PTSD decline. In LI, this process is called *trauma clearing* and is obtained using a specific protocol. In the present study we use a modified protocol, the Modified Lifespan Integration PTSD treatment protocol (MLI; WONSA, 2019). MLI was developed at the WONSA specialist clinic (WONSA SC) by developing and systemizing the original LI PTSD protocol into three different phases (*rapid exposure, cue jumping,* and *rescript*; Pace; WONSA, 2019). In summary, the method focuses on MCs. The MCs should be short (just a word or two are enough, for example “yellow bicycle” or “cinema”) and associated with negative, neutral or positive memories of everyday events. The MCs should be chronologically, evenly spread through the time span from the traumatic event to the present in a MC list. The more vivid the associated episodic memory of color, smell or other sensory detail is, the better. Normally 20–30 MCs are enough, regardless of whether the event took place several years ago or just a few weeks ago. The fragmented memories of traumatic event itself are retrieved during the phase of rapid exposure. The following cue jumping through the MC list is used to visualize time passed since the traumatic event and imaginary rescripting is used to replace shame with a sense of agency to the memory of the event. For further details of the intervention and possible explanations of its efficacy, please see the manual in the supplemental material.

The effect of LI on PTSD after a single sexual trauma has not been studied and only preliminary studies have been done in other patient groups. In Balkus’ (2012) outcome study, 17 women at a female residential treatment program in Seattle with different types of interpersonal traumas worked with one chosen index trauma for two sessions of LI. Changes in Impact of Event Scale (IES) were used as primary outcome. There was a major score reduction post treatment and further improvement was seen three months later (Balkus, 2012). In a case study, Hu analyzed LI on three patients with a history of childhood abuse. They received LI for three months. The results indicated that the participants experienced significant positive clinical change (Hu, 2014). Despite these positive results and anecdotal clinical experience (Catherine Thorpe, 2012) (Balkus, 2012; Hu, 2014), the effect of LI on symptoms of PTSD has not been examined in further clinical trials.

The aim of this study was to test the hypothesis that one session of MLI could reduce symptoms of PTSD (Jowett et al., 2019; Kolk, 2014; Rosenfield et al., 2018) as defined by *The Diagnostic and Statistical Manual of Mental Disorders* (DSM-5) and measured by IES-R (Jowett et al., 2019) among patients exposed to one single sexual assault, without earlier traumatization.

## Method

### Trial Design and Setting

This was a single-center, individually randomized waitlist-controlled treatment study with 1:1 allocation, conducted in Stockholm, Sweden at a specialist clinic for sexually traumatized patients (WONSA SC) between April 2016 and June 2019. Participants were included between April 26, 2016 and June 17, 2019. The clinic is run by the nongovernmental organization WONSA. All staff at the clinic are specially trained in trauma sensitive care and treating sexually abused patients. The authors assert that all procedures contributing to this work comply with the ethical standards of the relevant national and institutional committees on human experimentation and with the Helsinki Declaration of 1975, as revised in 2008. All procedures involving human patients were approved by the regional ethical review board in Stockholm (2015/1868-31/2).

(Study protocol is available at www.WONSA.org)

**Figure 1.**
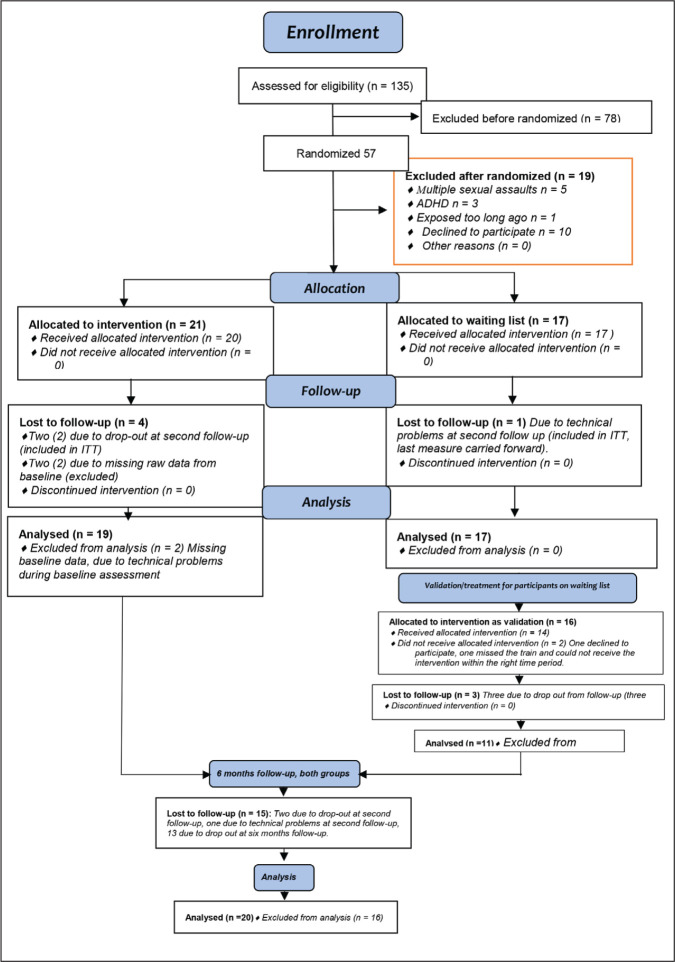
Consort 2010 flow chart of the study set-up.

Included participants were randomized to an intervention arm or to a waiting list arm. A baseline measure was made, after which the participants in the intervention arm were given the intervention. After the first follow-up after intervention/no intervention, the participants in the waiting list arm were given the same intervention as the participants in the intervention arm, with the same follow-up after the intervention. A third follow-up was conducted six months after the first follow-up (see Consort flow chart in Figure 1).

### Participants

Participants were recruited from the community via information on social media and contacted the clinic through e-mail address, telephone, or were referred to the clinic from other caregivers. Participants of any socioeconomic status, race, ethnicity, gender identity, sex, sexual orientation, religion or culture were welcome. We consecutively enrolled individuals who met inclusion and exclusion criteria according to self-report over the telephone or at the doctor’s office at the clinic. Written informed consent was obtained from all patients. Inclusion criteria were both sexes, age 15 and above, with one single sexual assault 0–5 years prior to inclusion. Exclusion criteria were poor understanding of Swedish, multiple traumas, active substance abuse, active psychosis, ADHD, or autism spectrum disorder as self-reported or assessed during the doctor’s visit. Informed consent was acquired in person or via online format. Self-rating was completed using an online questionnaire or on paper documents at the clinic (*n* = 2). To provide attention control to the control arm, all patients had a clinical assessment prior to the intervention. At this point patients with unreported complex traumatization, ongoing substance abuse or active psychotic episodes were also excluded. If excluded because of multiple sexual abuse, the patient was offered referral to psychiatric care or access to the intervention in the framework of the clinical setting instead at the specialist clinic.

### Interventions

**Intervention arm.** After inclusion, participants randomized to the intervention arm were given MLI in a single session. The MLI is a manual-based psychotherapy treatment (WONSA, 2019). Normally in clinical settings, the psychotherapist meets the patient in a 45-minute session before the MLI intervention and a follow-up after the intervention is also common. In this study, however, information about the intervention was given to all participants during the doctor’s visit. During the doctor’s visit the participants were also instructed to prepare a list of key episodic MC list. The MC list was asked to be prepared one or two days before the intervention.

With this procedure, the participants only met the psychotherapist during one 90–140-minute intervention, making the intervention as pure as possible. However, it is important to note that while the intervention took place during a single session, the intervention was preceded by a small but significant preparation, including time for information at the doctor’s visit, and the participant’s own work with writing the MC list before the intervention.

The MLI session itself begins with the patient and the therapist introducing themselves to each other. Thereafter, the therapist repeats information on the structure of the intervention. The patient explains the prepared MC list to the therapist, and then the intervention starts. (for more information about the intervention see supplemental material). The intervention was conducted by five different psychotherapists trained in MLI. Even though a safe attachment to the therapist is not hypothesized to be necessary when working with MLI, we wanted the therapeutic setting to be as safe as possible for the participants. Since the majority of perpetrators of sexual assault and rape are male, and just being alone in a room with a man can serve as a trigger for individuals with PTSD after sexual abuse, we chose to have only female therapists in this study. Four psychotherapists used rooms at the clinic and one had her treatment facilities outside of the clinic. The sessions were audio recorded and stored in order to make it possible to monitor the therapist’s adherence to the treatment protocol.

**Comparison arm.** Participants randomized to the comparison arm received an attention control in the form of the same doctor visit as the intervention arm at which they were clinically assessed, given information about the treatment, and asked to prepare a MC list one or two days before the offered intervention, after the second measurement at time point two. The participants in the waiting list arm also completed a third self-rating measurement 20 days (+/– 3) after the intervention. This design was chosen to minimize the time on waiting list for treatment and to allow validation of the results for the intervention arm.

### Randomization and masking

After inclusion, participants were randomized to intervention or waitlist control. Preparation of the trial material and randomization was conducted by the independent data and safety monitoring board Karolinska Trial Alliance (KTA) via sequenced computer-generated simple randomization with 1:1 allocation. Participants received sequentially numbered trial materials with concealed allocation. Allocation envelopes were kept and handled by the trial staff. Due to initial misunderstandings, the prepared sealed envelopes were initially not always picked in strict numeric order. However, the study was monitored and periodically reviewed by the KTA and the misunderstanding was corrected. Participants could not be blinded to their treatment allocation due to the nature of this nonpharmacological intervention. Therapists involved in delivery of the intervention were also unblinded. Since this was the first study of MLI in this context, we were concerned about risk for adverse effects and therefore planned external continuous monitoring of the results. We also initially planned blinded external statistical analysis. Due to a lack of resources, both the continuous monitoring of results and the analysis were done within the research team, without blinding.

### Outcomes

After inclusion and randomization, self-rating was completed by both arms at baseline (5 +/– 3 days before intervention or no intervention) and at time point two (20+/– 3 days after the intervention/no intervention). After measurement at time point two, participants in the comparison arm waited 5+/– 3 days and then received the same MLI intervention as in the intervention arm. Participants in the comparison arm completed a self-rating 20+/– 3 days after their intervention as a validation of the effect size in the treatment group. All participants were also contacted for a follow-up six months later.

**Primary outcome.** The primary outcome was the difference between the two trial arms in mean PTSD symptoms as measured by the Impact of Event Scale Revised (IES-R) at time point two. The IES-R has shown high internal consistency and the correlation between the IES-R and the PTSD checklist (PCL5) has been shown to be high (Creamer et al., 2003; Murphy et al., 2017). A cut-off for PTSD at 34 points has provided good sensitivity (0.86 and 0.89 respectively *n* = 854 and *n* = 3,313) in large studies, and the findings support the use of IES-R in studies of PTSD when diagnostic interviews are regarded as too costly or labor intensive to conduct (Morina et al., 2013). In this study the cut-off at 34 points is being used, and scores with a minimum of 34 points will be referred to as “scored as PTSD”.

**Secondary outcomes.** To verify the results on IES-R, the National Stressful Events Survey PTSD Short Scale (NSESSS) was used as a secondary outcome scale (LeBeau et al., 2014). NSESSS has only nine items, but has shown high internal consistency with DSM-5 diagnostic criteria (LeBeau et al., 2014). Other secondary outcomes were differences between the two trial arms in the General Health Questionnaire (GHQ12), measuring nonspecific psychological distress at time point two (Goldberg & Williams, 1988). The GHQ12 has been used multiple times over the years in the public health questionnaires in the Stockholm Region, enabling comparison of psychological health in our study population with that of the general population. To compare the general health before and after the MLI, we requested data from the Stockholm Region survey on the health in the population on GHQ12 of 2018 in women of the same age group as the participants of the present study.

### Sample Size, Interim Analysis and Stopping Guidelines

Our primary objective was to detect a difference in mean symptoms of PTSD at time point two between the intervention and comparison arms. In order to detect a standardized effect size of *d* = 0.7 (80% power, 5% significance level for a two-sided test), we required 33 participants per trial arm. Allowing this with 40% attrition resulted in a total target sample size of 100 participants. Because this was the first randomized controlled treatment study conducted for the method, we followed the results in order to be able to detect adverse effects (i.e., elevated scores on self-rating at time point two). The major differences between the groups at time point two in combination with lack of funding, led us to the decision to perform an interim analysis when 36 patients had completed the second measurement at time point two. An extremely large effect size far above the estimated *d* = 0.7 on which we had estimated the original number of participants was found and the study was closed.

### Statistical Methods

An intention to treat (ITT) approach, in which all participants are analyzed in the study arm to which they were randomly allocated, was applied to the analyses for primary and secondary outcomes. Differences in mean outcomes between intervention and comparison arms at time point two were analyzed using analyses of covariance (ANCOVAs), using baseline scores as covariates. Cohen’s d was calculated as a measure of between-group effect size in the intention to treat analysis, where 0.2 is regarded as a small effect, 0.5 a moderate effect and 0.8 a large effect. Due to multiple tests conducted, a *p* value of less than .05 was regarded significant for our primary outcome IES-R, and .01 was regarded significant for the secondary outcomes NSESSS and GHQ12. Stata version 14.2 was used.

### Ethical Considerations

All data handled were coded and none of the individuals could be identified in the datasets from the study. Ethical approval was obtained from the regional ethical review board in Stockholm (2015/1868-31/2). The trial was given registration number NCT03141047 at ClinicalTrial.gov in March, 2016.

## Results

### Participants

Among the 135 individuals who contacted the clinic between April 2016 and June 2019 for participation, 57 were eligible, 100% were women, age 15–65 years, median age 24 years. All participants were Caucasians. At the intake meeting with the doctor, another 21 patients were excluded. Of the 21 patients excluded after the doctor’s visit, five had multiple sexual traumas, three had diagnoses of ADHD, one had the index trauma more than five years ago, and ten patients declined to participate, and two had missing data from baseline measures. Of the remaining 36 participants, all were included in the intent to treat analysis. However, two could not complete the second self-rating because of technical problems with the online self-rating, and one participant forgot to answer within the given time frame. The remaining 33 participants all completed the second self-rating, and were also analyzed per protocol for the primary outcome (Table 1) (CONSORT 2010; Figure 1).

**Table 1.** Comparison Between the Intervention and Waiting List Arm at Time Point Two, Twenty Days After One MLI Session or Twenty Days After No Treatment (On the Waiting List Arm).

	**Intention to Treat Analysis**					**Per Protocol Analysis**		
	MLI (n = 19)		Waiting list (n = 17)	F-value	Difference*p* value	MLI (n = 17)	Waiting list (n = 16)	Difference *p* value
	Mean	Effect size						
IES-R	24.7 (16.4)	2.43	55.2 (15.5)	21.37	<.001	22.2 (15.6)	56.6 (14.9)	<.001
NSESS	8.5 (6.6)	1.80	19.6 (5.4)	22.90	<.001	7.6 (6.4)	19.9 (5.5)	<.001
GHQ12	14.2 (6.9)	1.11	20.2 (5.7)	10.93	.0013	13.4 (6.6)	20.8 (5.3)	.0003
PTSD*						71%	6%	

*Note.* *34 points or above on IES-R. When data was missing at time point two the last observation (baseline investigation data) was carried forward in the intention to treat analysis. Subjects with missing data (n = 3) were excluded in the per protocol analysis. ANCOVA adjusted for the baseline measurements was used to calculate *p* values.

### Baseline Characteristics

Characteristics of the study sample before treatment was collected at baseline (Table 2). There were no significant differences in means between the two groups before treatment with the MLI. The medium IES-R scores were 58.0 (SD 13.7) and 55.2 (SD 14.1) in the two arms respectively. 95% (18 out of 19) of the participants in the intervention arm and 88% (15 out of 17) of the participants in the waiting list arm scored for PTSD at baseline. The medium GHQ12 scores were high: 20.7 and 20.1 in the two arms, respectively. The average age in both treatment arms was 24 years.

**Table 2.** Characteristics of Study Sample in Those Randomized to MLI and Waiting List at the Baseline Investigation. Means (Standard Deviation) are Presented.

	MLI (n = 19)	Waiting List (n = 17)	Difference*p* value
Age, years	24.2 (6.3)	24.2 (6.3)	.99
Months since rape	31.7 (16.4)	23.6 (17.6)	.18
Impact of event scale (IES)	58.0 (13.7)	55.5 (14.1)	.60
NSESS	20.7 (6.8)	20.1 (5.7)	.77
General health questionnaire 12 (GHQ12)	22 (7.0)	20.9 (5.3)	.60

### Intention to Treat Analyses

**Primary outcomes.** The estimated between-group effect size in the intention to treat analysis was *d* = 2.43 (Table 1) in favor of MLI and 72% of the patients in the per protocol analysis no longer scored as PTSD after the MLI one session treatment (Table 1) in contrast to only 6% in the control group. The intention to treat analysis between time points one and two within groups are shown in Table 3. The IES-R (F = 21.37, *p* < .001) scores were on average halved in the intervention arm, and essentially unchanged on the waiting list arm. The six-month follow-up for both groups are shown in Table 4. The IES-R remained stable at the six-month follow-up.

**Table 3.** Intent to Treat Analysis of Points on Self-rating Scales At Time Points One and Two.

	**MLI (n = 19)**			**Waiting List (n = 17)**		
	Time 1	Time 2	*p* value	Time 1	Time 2	*p* value
Impact ofevent scale	58.0 (13.7)	24.7 (16.4)	<.001	55.2 (14.1)	55.1 (15.5)	.92
NSESSS	20.7 (6.8)	8.5 (6.6)	<.001	20.1 (5.7)	19.6 (5.4)	.73
General health questionnaire 12	22.0	14.2	<.001	20.9 (5.3)	20.2 (5.7)	.61

*Note.* Results by paired t-tests.

**Table 4.** Per Protocol Analysis at Six Months Follow-Up After MLI-Treatment in 14 MLI patients, and at Six Months Follow-Up After Treatment for 6 Waiting List Patients. (WMLI).

	MLI (n = 14)			WMLI (n = 6)		
	Time 2	Time 3	*p* value	Time 2*	Time 3	*p* value
Impact of event scale	19.9 (14.9)	17.9 (14.8)	.5	26.2 (14.6)	22.2 (17.3)	.25
NSESSS	6.6 (5.6)	6.7 (7.7)	.0	8.2 (1.1)	6.7 (2.1)	.6
General health questionnaire 12	12.7 (6.6)	12.1 (7.8)	.7	13.2 (6.2)	16.3 (10.3)	.5

Note. Results by paired t-tests.

*Measurement after treatment for waitinglist patients.

**Secondary outcomes.** NSESSS (F = 22.90, p < 0.001) scores were on average halved in the intervention arm, and essentially unchanged on the waiting list arm. The GHQ12 (F = 10.93, *p* < .001) scores were more moderately reduced in the intervention from 20.0 to 14.3, but still rendered a between-group effect size in the intention to treat analysis Table 3. A per protocol validation in 11 patients on the waiting list, that were taking part in MLI after time point two and filled out their ratings, is shown in Table 5. Among these patients 82% lost their scored PTSD (minimum 34 points at the IES-R), average scores on IES-R and NSESSS were halved (*p* < .001 for both). The score for GHQ12 was more moderately reduced. The six-month follow-up for NSESSS and GHQ12 are shown for both groups in Table 4. Both NSESS and GHQ12 remained stable at the six-month follow-up.

**Table 5.** Per Protocol Validation after MLI Treatment in 11 Waiting List Patients.

MLI in Waiting List Arm			
	Time 2	Time 3	*p* value
Impact of event scale	58.4(14.7)	23.1(13.0)	<.001
NSESSS	21.5(3.7)	9.3(5.4)	<.001
General health questionnaire 12	20.8(5.7)	14.9(7.6)	.076

*Note.* Results by paired t-tests.

### Harms

No harms were detected (i.e., there were no elevated scores on IES-R, or NSESSS at time point two or three).

## Discussion

The main finding was that MLI given as a one-session treatment, gave efficacious PTSD symptom reduction at three-week follow-up compared to a waiting list control and remained stable at the six months follow-up. The intervention arm showed reduced IES-R and NSESSS scores by half at three-week follow-up. The waiting list control arm had unchanged scores in analyses by both per protocol and by intention to treat. Moreover, 72% of the patients in the per-protocol analysis no longer scored as PTSD (< 34 points at the IES-R) after the MLI one-session treatment, compared to 6% or the participants on the waiting list.

Another finding was the significant reduction of psychological distress, as measured by GHQ, among the participants in the trial arm.

Finally, the changes in scores between baseline and time point two (and three in the waiting list arm) on IES-R and NSESSS followed the same pattern, indicating congruence with the PTSD definition of DSM5. The per-protocol analysis after the treatment of the 11 patients randomized to waiting list who completed the post treatment self-rating at time point three, showed similar improvement after having been given the same intervention compared to those in the treatment arm, indicating that the results are robust.

When comparing the results from this study with prior PTSD treatment studies, one must keep in mind that in this study, we included only PTSD after sexual assault at one single occasion. This is a narrower inclusion criterium than in previous studies where different types of traumas may have been included and were no distinction has been made neither between repeated and single trauma nor between PTSD and complex PTSD at inclusion. The large effect size in this study may partly be due to this narrow inclusion criterium. As true as this is, it is also a fact that hitherto, PTSD treatment studies normally include 8–12 sessions, and the results from this study are comparable to the results of PTSD treatment from 8–12 sessions studies of PE, CR, trauma-focused CBT as well as for EMDR and ImRs, but with only one single session and with a minimal dropout rate: In a randomized trial of PE for treatment of adult rape survivors (*n* = 47) (Foa et al., 1991), mean PTSD symptom reduction after 9–12 sessions with PE treatment at first follow-up (PTSD symptom scale interview) was 45.9%, compared to 57.4% in our study. In another randomized trial of PE for treatment of sexual abuse-related PTSD in adolescent girls (*n* = 31; Foa et al., 2013), a 65.4% symptom reduction of self-rated PTSD was seen after 14 90–120-minutes sessions of PE, compared to 57.4% symptom reduction in our study. In the same study 78.4% of the participants did not score as PTSD after the study, compared to 72.2% of the participants in our study. In a meta-analysis from 2013, including 42 studies on PTSD treatment, the average dropout rate regardless of intervention was 18% (Imel et al., 2013).

The single session design in this study minimized rates of discontinued intervention to 0% (*n* = 0). Even if MLI treatment improved general health as measured by the GHQ12 significantly in the present study, there is still room for improvement to reach the general health level of the average woman in the Region (Folkhälsomyndigheten, 2018). The GHQ12 score among 24-year-old’s in the Stockholm Region was in comparison 11.7 (Folkhälsomyndigheten, 2018; data not shown in tables).

The long recruitment period might partly be explained by the narrow inclusion criteria, but was also in part expected based on the ambivalence to disclosure and treatment among victims of sexual abuse (Collin-Vezina et al., 2015; Patterson et al., 2009). It is also reasonable to assume that the method in the study being new, with no prior published results, further increased preexisting ambivalence.

The fact that 33% of the individuals contacting the clinic for inclusion reported multiple sexual abuse *despite* information about inclusion criteria in the advertisement of the study, might partly be a result of study information being spread through a short ad through social media, were details might not always be paid attention. Second, it is also plausible that inclusion criteria was overlooked by individuals having difficulties finding PTSD treatment after sexual abuse, considering the gap between the need and the access to nonemergency healthcare services for victims of sexual abuse in Sweden (SKR, 2020).

One could argue that the long recruiting period for patients with single sexual assault and the big number of patients seeking help for multiple abuse, indicate the method tested have no “real world” value, outside the research setting. However, the fact that individuals with experience of one single rape or similar single sexual trauma are reluctant to seek help, does not mean they do not need help. On the contrary, rape is the trauma with the strongest correlation to the development of PTSD of all known traumas, and revictimization is common (Ranjbar & Speer, 2013). Furthermore, earlier studies have shown rape victims tend not to seek help because of fear of not being believed, or being offered interventions that will not helpful (Patterson et al., 2009). With this perspective the long recruiting period rather highlights the importance of finding helpful interventions for first time sexual abuse victims, than the opposite. Being early stage research, strict and narrow inclusion criteria was prioritized in this study, in order to lay the foundation for future studies with broader inclusion criteria. The method is already being used for different types of single traumas, and with additional protocols for multiple traumas as well as for complex PTSD (CPTSD; Rosenfield et al., 2018), but further studies are needed.

The need to distinguish between PTSD and CPTSD has been argued since Judith Herman first proposed the diagnoses in the 1980s. To make this distinction when evaluating trauma treatment is more relevant than ever considering CPTSD has now been added to the ICD 11 (Rosenfield et al., 2018), opening up for perspectives that might allow more efficient healthcare processes and patient flows to be evaluated.

### Strengths

The well-defined inclusion criteria, that the intervention was given by different therapists and the opportunity to test the per protocol effect of the MLI after the second self-assessment at time point three, in participants on the waiting list as a validation of the effect size, are strengths. Another strength is symptom rating with two different tools for assessing PTSD, IES-R and NSESSS were congruent. In addition, the comparison arm received an attention control in the form of a doctor’s visit with information about the intervention including instructions for preparing the MC list.

### Weaknesses

This study has several weaknesses, many related to a lack of resources and the need to use the most cost-effective procedure as possible. This affected both inclusion, randomization, and the sample size: Inclusion and randomization after the clinical assessment previous to attention control would be preferable, as would a larger sample. Other limitations associated with resources were that allocation envelops were kept and handled by the trial staff and that we relied on self-report data and self-rating rather than on clinical assessment. We used a waiting list design with a very short follow-up period, both factors that can exaggerate the efficacy of the intervention (Cunningham et al., 2013). It is possible that the attention control given to the comparison arm could lead to bias in that receiving information about the intervention and instructions for preparing the MC list could lead symptoms to worsen; however, we felt it was necessary to give an attention control to this group in order to examine only the effect of the single session MLI. To minimize this possible bias, the information given at the attention control was very limited. The self-ratings remained stable between time points one and two for the control group, indicating that the limited information given at the attention control did not affect primary outcome in any substantial way. Our waiting list design, with treatment for the waiting list arm five days after the self-rating at time point two, did not allow between-group analysis of long-term follow-up.

Another weakness is that no data on socioeconomic status, gender identity, sexual orientation, religion or culture was collected in this pilot study.

However, neither the narrow inclusion criteria, waiting list bias, nor short follow-up time are likely to completely explain the estimated between-group effect size in the intention to treat analysis in this study.

### Diversity

Any individual age 15 and above who met the inclusion criteria was welcome. No questions about socioeconomic status, gender identity, sexual orientation, religion or culture were asked. All participants were Caucasians. Diversity was not further explored in this pilot study.

## Clinical Implication

MLI is a promising one-session treatment for PTSD in women after a single sexual assault. Studies comparing MLI to best practice PTSD treatments in individuals with PTSD, but not CPTSD, with a larger sample size and longer follow-up are warranted. The results indicate an important treatment effect and a low dropout rate. Provided that the results from this study are replicable beyond the per protocol validation in the present study, in other settings and for other single traumas than sexual assaults, the developed method is likely to be less demanding for patients and less costly for society than traditional treatments.

## Supplemental Material

Supplemental material for A One-Session Treatment of PTSD After Single Sexual Assault Trauma. A Pilot Study of the WONSA MLI Project: A Randomized Controlled Trial Click here for additional data file. Supplemental material for A One-Session Treatment of PTSD After Single Sexual Assault Trauma. A Pilot Study of the WONSA MLI Project: A Randomized Controlled Trial by Gita Rajan, Caroline Wachtler, Sara Lee, Per Wändell, Björn Philips, Lars Wahlström, Carl Göran Svedin, and Axel C. Carlsson in Journal of Interpersonal Violence

## Author Biographies

**Gita Rajan**, MD, PhD candidate, specialized in Family Medicin. Dr Rajan is also the founder of the NGO World of No Sexual Abuse (WONSA). The vision of WONSA is a world of no sexual abuse and the mission is to increase the access to efficient health care for victims of sexual abuse.

**Caroline Wachtler**, MD, PhD, specialized in Family Medicin. Her clinical work as a resident in primary care (Stuvsta Primary care Center AB since 2009) informs her interest in mental health as she is daily made aware of the gap between research and practice. Caroline boasts a rare combination of skills in both qualitative and quantitative methodologies.

**Sara Lee**, Psychotherapist, specialized in cognitive behavioral therapy. Lee did her master’s degree from Svenska Kognitiva Institutet, Stockholm. Sara is working as a therapist at WONSA.

**Per Wändell**, MD, PhD, specialist in family medicine, senior professor of family medicine at Karolinska Institutet, former director of the Research School of Family Medicine at Karolinska Institutet. Research mainly on diabetes and cardio-vascular diseases in primary care, and of migration medicine.

**Björn Philips**, PsyD, is a psychologist and associate professor of psychology at the Stockholm University. Dr Philip´s main area of research and teaching is psychodynamic therapy, especially contemporary variants such as mentalization-based therapy, affect-focused therapy, and relational psychotherapy, and he is experienced on treatment studies.

**Lars Wahlström**, MD, PhD, specialized in Psychiatry, is an expert in consultation liaison psychiatry at Stockholm Health Care Services and clinician at the CL Unit, Psykiatri SV at Karolinska University Hospital Huddinge and doctor of psychiatry. Formerly at Crisis and Disaster Psychology Unit at the Centre for Family Medicine.

**Carl Göran Svedin**, MD, PhD, specialized in Child and adolescent Psychiatry, senior professor at at Ersta Sköndal Bräcke University College, Stockholm. Professor Svedin´s research has covered areas such as child physical and sexual abuse, sexual exploitation, children exploited in child pornography, prostitution. He has been responsible for three previous Swedish epidemiological studies on young people’s sexuality and sexual abuse (2004, 2009, 2014) and now carrying out the fourth.

**Axel C. Carlsson**, PhD, associate professor, pharmacologist. Skilled in biomarkers, epidemiology, and molecular biology. He is principal for the research school in family medicine and primary care. He is also working as investigator at the Swedish Public Health Agency.
