# Supplementary material for: A One-Session Treatment of PTSD After Single Sexual Assault Trauma. A Pilot Study of the WONSA MLI Project: A Randomized Controlled Trial
Source: J Interpers Violence. 2020 Oct 21;37(9-10):NP6582–603. doi: 10.1177/0886260520965973 (PMC9092905; doi:10.1177/0886260520965973)
Supplement: Supplemental material for A One-Session Treatment of PTSD After Single Sexual Assault Trauma. A Pilot Study of the WONSA MLI Project: A Randomized Controlled Trial [file sj-pdf-1-jiv-10.1177_0886260520965973.pdf]

# Manual for the Modified Lifespan Integration PTSD protocol: MLI

# Table of Content


**Manual for the Modified Lifespan Integration PTSD protocol: MLI....1**

***Introduction*....3**

***Lifespan Integration and PTSD*....3**

***A manual based intervention*....4**

***Who should be treated, and who can perform MLI?*....4**

***Vocabulary of MLI*....5**

***Preparation*....6**

Psychoeducation....6

Episodic Memory Cues List (MCs and MC-list)....7

*Phase one: Rapid Exposure ....7*

*Phase two: Cue Jumping and Timeline Repetition....9*

*Phase 3: Re-script and Timeline Repetition....9*

***Possible Explanations of the Efficacy of MLI*....11**

## Introduction

Lifespan Integration was originally developed by the social worker and psychotherapist Peggy Pace in 2002 -2012, when she noted she could speed up recovery from traumatic events, by helping her patients to identify and visualize key episodic memories (MCs) for each year since the traumatic event up to present time. In the book *"Lifespan Integration: Connecting Ego States through Time"* (2007 and 2012) Peggy Pace describes the method. In 2012 M.A Catherine Thorpe describes the further development of Lifespan Integration and its different protocols, in a second book, *"The success and Strategies of Lifespan Integration".*

## Lifespan Integration and PTSD

In the theory of Lifespan Integration, it is hypothesized that: *The core of the PTSD symptoms (intrusion, hypervigilance and avoidance), are due to a failure of the index trauma to anchor as an episodic memory in the person´s chronologic autobiography.* As a consequence, fear extinction is not used as a construct in the theory, but *fear memory transformation* (from a defragmented traumatic memory to an episodic memory) *and episodic memory anchoring and integration* (from a dissociated episodic memory to an integrated episodic memory, anchored in a chronological time line of the autobiography). Accordingly, the symptoms of PTSD are neither seen as pathologic or dysfunctional cognitions or fear associations, nor as distorted beliefs as suggested in earlier studies (1-3). The symptoms are rather seen as logic responses to a failed memory construction: Without time anchoring, the limbic system interprets the index trauma not as a terminated earlier life-threatening event that the person has survived, but as a possible threat that needs to be handled in present time. With this perspective, intrusion and hyper vigilance are appropriate and lifesaving processes, and avoidance an effective solution to a neuro-biological conflict between the prefrontal cortex´ cognitions about the index trauma, and the limbic system´s interpretation, of the same trauma.

Subsequently, as the index trauma is transformed into an episodic memory anchored in a chronological time line, the limbic system stops perceiving the index trauma as a potential threat in present time. As a consequence, intrusion and hypervigilance stops, avoidance is no more needed, and the cardinal symptoms of PTSD* declines. In Lifespan Integration, this process is called *Trauma Clearing*.

## A manual based intervention

The MLI is a strict manual based intervention, carried out in one 90-120 minutes single session. The MLI manual is developed for treatment of PTSD. For complex traumatized (CPTSD) patients and patients with developmental traumas (DTD), other protocols are more suitable. For patients who have been unconscious during the trauma, or during parts of the trauma, body work is recommended to be added to the treatment. The MLI is developed from the Lifespan Integration PTSD-protocol (4). The aim of the MLI is Trauma Clearing as in the original PTSD-protocol, and to add a sense of agency and compassion to the reaction of the traumatic event. In clinical practice the patient usually meets the therapist for one 45 minutes session before starting the MLI, and a follow up session after the treatment is also common. In this manual, we focus on the specific MLI intervention, during one single session.

## Who should be treated, and who can perform MLI?

The MLI is constructed for treatment of PTSD after one specific index trauma. During the intervention the therapist and the patient work together with three different phases:
Rapid Exposure, Cue Jumping & Timeline Repetition and Re-script & Timeline Repetition.
The therapist giving MLI treatment must be adequately trained in the method. Training by certified LI-instructors or MLI trainers is recommended.

*DSM4

## Vocabulary of MLI

1. Fear Memory Transformation: The process of turning defragmented details of a traumatic event, in to an episodic traumatic memory during Rapid Exposure

2. Episodic Memory Anchoring & Integration: The process of transforming an episodic traumatic memory in to an integrated episodic memory, anchored in the patient's chronological autobiography, during Cue Jumps and Timeline Repetition.

3. Memory Cue (MC): A short sentence or word, associated to a specific episodic memory.

4. Memory Cue List (MC-list): A list of MCs, put in a chronological order. The list starts in past time, and ends in present time.

5. Rapid Exposure: The process of rapid chronological retrieving of defragmented details of a traumatic event. The process is guided by the therapist, who moves the patient forward in time through the traumatic event by asking: *"And then, what happened?"* Speed Affect Regulation is used to time the repeated question.

6. Cue Jumping: The process where the therapists reads MCs from the MC-list to the patient. Speed Affect Regulation is used to time the Cue Jumps.

7. Speed Affect Regulation (SAR): A method were the therapist helps the patient to regulate emotions by adjusting the fractions of seconds of memory re-experiencing, and the speed in which the patient is moved from the past to the present along the MC list.

8. Round: The procedure when the therapist reads the MCs and guides the patient from the first to the last MC.

9. Timeline Repetition: The repetition of rounds.

10. Trauma Clearing: The process in which the patient's chronological autobiographic memory is cleared from non-anchored traumatic memories. When a trauma is cleared, there should be no re-experiencing of traumatic memory details when recalling the memory, or when exposed to details associated to the traumatic event. Trauma clearing is the goal of the MLI.

## Preparation

*Psychoeducation*

It is important that the patient understands how the intervention is carried out, and the purpose of the various phases in the treatment. Psychoeducation of how symptoms of PTSD are explained by the Lifespan Integration theory is therefore obligatory before starting MLI. A schematic drawing of "normal" anchored episodic memories as compared to traumatic non-anchored memories might be helpful. Before starting the treatment, make sure:

- There is enough time for the full treatment to be completed – a minimum of 90 minutes is required.

- That the patient understands the importance of carrying the intervention on until the end. If the session is stopped before the trauma is cleared, (i.e. the memories of the trauma is activated without being anchored in the chronological autobiography) the symptoms of PTSD will rise instead of decline.

- That the patient understands that affect regulation will be performed through SAR: If emotions get intense, the therapist will *speed up* the transition through the timeline, not slow down the process.

- That no place will be given for confirmation or comfort of emotions during the rounds. Neither will place be given for reflections about the event itself, or for reactions or cognitions about the event during the rounds. It is important to inform the patient that the manual is constructed in this way in order to make the symptoms of PTSD to decline as quick as possible. When the trauma is cleared, confirmation and reflection can be done with less emotional pain.

*Episodic Memory Cues List (MCs and MC-list)*

In the next step, information about how to make an MC-list is given. Every MC must give an association to a real **episodic memory, anchored in time.** It does not matter whether the episodic memory associated to the MC is negative, neutral or positive. The more vivid (if the patient recalls colors, smells or other sensory details of the chosen memory) the associated episodic memory is, the better. The MCs should be short, just a word or two is enough, for example *"yellow bicycle"*, or *"cinema"*. They should be put in a chronological order, creating a timeline from just before the traumatic event to the present. Normally 20-30 MCs are enough. The MCs should be spread evenly through the time span from the traumatic event to the present, whether the event took place several years ago or just a few weeks ago. **The traumatic event itself, should not be put on the MC-list before the session.** The MCs from the traumatic event are created by therapist, during the Rapid Exposure Phase. The MC-list is preferable made by the patient before the MLI-treatment. If it is difficult for the patient to do the MC-list at home, it can be made together with the therapist during a session before the MLI treatment. Not being able to do the MC-list independently might however be a sign of a more complex PTSD, and is a reason for further assessment.

## Phase one: Rapid Exposure

The technique of Rapid Exposure serves two different purposes: 1) To activate the Hippocampus through retrieval and a light re-experiencing of the defragmented details of the trauma and: 2) To help the patient describe important details of the trauma in a chronologic time order. The therapist takes notes during the Rapid Exposure, and turns them into MCs on the MC-list in the coming rounds. The therapist starts the Rapid Exposure phase by asking the patient to describe what the patient was doing one or two weeks before the index trauma. This will be the 1 st MC, and it serves to link the patient to life as it was before the index trauma.

Then the therapist asks what the patient was doing just before the traumatic event. This will be the second MC. After this second MC, the therapist asks the patient to describe the first remembered detail of the traumatic event, and to continue to tell about the traumatic event in a chronologic order. During this phase the therapist takes notes and creates the rest of the MC list of the traumatic event. All trauma details should not be written on the MC list since the MCs list must be possible to repeat for 10-20 rounds during the session in order for Trauma Clearing to take place. It is also good to leave spaces in the MC list created in this phase. The spaces can be filled in later (between the rounds), as more memories of the traumatic event often come up during the following rounds. If the patient stops in the middle of the trauma story, the therapist helps the patient to move forward by asking: "Then what happened?" To minimize re-experiencing, Speed Affect Regulation (SAR) is used. In practice, SAR means that the therapist helps the patient to regulate the arousal, by moving the patient through the traumatic event by asking "and then what happened" as soon as there are signs of body reactions related to the re-experience of the retrieved defragmented traumatic memory details (faster breathing, body tension etc). Nota Bene: It doesn't matter if the next cue is worse than the previous one, as long as the patient moves forward, through all the MCs of the trauma, and into present time. It is the chronological movement from one detail of the trauma to the next that is hypothesized to induce the Fear Memory Transformation. Even though no time for reflection or emotional processing is given in this phase, it is still critical that the therapist stays attuned to the patient during the process. The errors of the chronologicity of the MCs created in this phase, is corrected in the coming rounds. Rapid Exposure is conducted only once, and this is the only time the patient needs to talk about the index trauma.

## Phase two: Cue Jumping and Timeline Repetition

The aim of Phase 2 is also twofold: 1) To turn the defragmented details of the trauma in to an episodic memory, and: 2) To anchor the new episodic memory to the chronologic time line of the autobiography of the patient. The phase starts immediately after the last detail of the traumatic event has been described in Rapid Exposure. The therapist asks the patient to close the eyes (if the patient is comfortable with doing that, otherwise they continue with eyes opened) and the first MC of the pre-prepared MC-list of MCs, from the time immediately after the traumatic event to the present, is given. All the **MCs should be given in present tense.** The patient is asked to visualize the MCs. When visualized the patient gives the therapist a sign (lifting a finger is often used). Each time the patient has given a sign of visualization, a new MC is given by the therapist until present time is reached, and one round is completed. After a short break, next round starts. From the second round and on, the therapist starts by using the MC list created during the Rapid Exposure, and continues with the pre-prepared MC list. Again, SAR is being used for affect regulation as the patient is moving through the MCs of the traumatic event. This time however the therapist does not ask "What happened then?", but is instead reading the next MC on the MC list. No time for reflection or emotional processing is given during the timeline repetition. When the cue jumping can be made in a slower pace without high arousal all the way through the traumatic event, normally after 10 – 20 rounds, the therapist starts the third phase.

## Phase 3: Re-script and Timeline Repetition

The aim of the last phase is to enhance and integrate the feeling of agency and compassion in relation to the index trauma, in to the autobiography. During this phase the therapist helps the patient to re-enter the traumatic event as the older self (it does not matter if the patient is only a few days older or several years older at present time, compared to the age at the time for the

traumatic event). The therapist then helps the older self to help the younger self to find alternative imageries of actions or solutions to the traumatic event. In this process imageries of not being alone, of anger being expressed or actions are taken to defend themselves, to punish the perpetrator or simple to call for help or to be comforted are useful. (During the break before phase three is started, the therapist and the patient can discuss how the patient wishes to be helped during the phase). After the imagery, the older self takes the younger self to an imagery place where both the older and younger self feels safe. The older self tells the younger self that she/he has survived, that the traumatic event is terminated and that she/he will now be showed what has happened since then. After this, cue jumping from the first MC after the traumatic event, to the present time, starts again. Depending on the needs of the patient, the following rounds are conducted with or without the imagery re-scripting part. The session is completed when all the cue jumps, including the details from the traumatic event, can be performed in a calm, slow pace, without arousal or affect activations and without disturbing sensations in the body.

## Possible Explanations of the Efficacy of MLI

The practice of Rapid Exposure, with a fast and structured retrieval of defragmented details of the index trauma, is hypothesized to catalyze the start of the episodic memory construction, through Hippocampal activation. A process hypothesized to be completed by the following Cue Jumping. This theory is in line both with EPT and the hypothesis that activation of fear structures are needed in order to increase plasticity in the memory structure (2), and the findings that imaginal exposure does not need to be as long as in traditional PE (5, 6). The theory is also in line with research and theories exploring stress reduction during treatment as an important factor for new learning and restructuring (7).

The practice and theory of Cue Jumping and Timeline Repetition is hypothesized to have *two central components* contributing to the efficacy of MLI. Even though the theory of Lifespan Integration, as presented here, does not use fear extinction, but fear memory transformation and anchoring, the *first central component* is in line with animal research on fear extinction and new learning, where the proximity in time between retrieval and destabilization of fear memories and novelty introduction, were associated with the transfer of short-term extinction memory to long-lasting memory and reduction of fear reinstatement (1, 8). In the theory of rapid exposure and cue jumping, the transformed fear memory (i.e. the newly retrieved and transformed defragmented details of the index trauma to an episodic memory of an earlier terminated event), can be understood as a form of fear extinction. The following timeline repetition through cue jumping, where the new episodic memory is anchored in time, can be seen as a way of introducing novelty adjacent to the "extinction" process. *The second central component*, is in line with earlier research on how auditive and visual areas in the brain are activated whereas speech areas are shut down, when exposed to traumatic stress (9). By listening and visualizing the chronological MCs given by the therapist during the cue jumping, it is hypothesized that the brain areas associated with speech are bypassed. Allowing

more active and therefore plastic networks to be used during the timeline repetition, is thought to speed up the process of consolidation, anchoring and integration of the new episodic memory (i.e. the core of the Lifespan Integration theory).

The practice of Imagery Re-script and Timeline Repetition is in line with the importance EPT gives to processing of negative trauma-related cognitions(2). However, instead of using exposure, habituation and reflection as means for cognitive restructuring, imagery re-script is used to induce a new felt experience of agency and compassion as the bas for a new cognitive concept.

In summary, the use of Rapid Exposure to induce the creation of an episodic memory, the use of Imagery Re-script to create a new cognitive concept and the use of auditive and visual Timeline Repetition to consolidate, anchor and integrate the new episodic memory and the new cognitive concept to the chronologic autobiography, is hypothesized to explain the fast decline of PTSD symptoms in MLI.

## References


1. Kida S. Reconsolidation/destabilization, extinction and forgetting of fear memory as therapeutic targets for PTSD. Psychopharmacology (Berl). 2019;236:49-57.
2. Foa EB, McLean CP. The Efficacy of Exposure Therapy for Anxiety-Related Disorders and Its Underlying Mechanisms: The Case of OCD and PTSD. Annu Rev Clin Psychol. 2016;12:1-28.
3. Cooper AA, Zoellner LA, Roy-Byrne P, Mavissakalian MR, Feeny NC. Do changes in trauma-related beliefs predict PTSD symptom improvement in prolonged exposure and sertraline? J Consult Clin Psychol. 2017;85:873-882.
4. Pace P. Lifespan Integration PTSD-Protocol.
5. van Minnen A, Foa EB. The effect of imaginal exposure length on outcome of treatment for PTSD. J Trauma Stress. 2006;19:427-438.
6. Nacasch N, Huppert JD, Su YJ, Kivity Y, Dinshtein Y, Yeh R, Foa EB. Are 60-minute prolonged exposure sessions with 20-minute imaginal exposure to traumatic memories sufficient to successfully treat PTSD? A randomized noninferiority clinical trial. Behav Ther. 2015;46:328-341.
7. Maren S, Holmes A. Stress and Fear Extinction. Neuropsychopharmacology. 2016;41:58-79.
8. Liu JF, Yang C, Deng JH, Yan W, Wang HM, Luo YX, Shi HS, Meng SQ, Chai BS, Fang Q, Chai N, Xue YX, Sun J, Chen C, Wang XY, Wang JS, Lu L. Role of hippocampal beta-adrenergic and glucocorticoid receptors in the novelty-induced enhancement of fear extinction. J Neurosci. 2015;35:8308-8321.
9. Kolk vd: in The Body Keeps the Score, Chapter 3 Looking into the Brain: The neuroscience revolution, The Penguine Groupe; 2014.
